# Epithelial Cell-Neutrophil Interactions in the Alimentary Tract: A Complex Dialog in Mucosal Surveillance and Inflammation

**DOI:** 10.1100/tsw.2002.77

**Published:** 2002-01-09

**Authors:** Sean P. Colgan, Katrina M. Comerford, Donald W. Lawrence

**Affiliations:** Center for Experimental Therapeutics and Reperfusion Injury, Brigham and Women's Hospital and Harvard Medical School, Boston, MA 02115, USA

**Keywords:** mucosa, infection, inflammation, integrin, eicosanoid

## Abstract

Inflammatory diseases of mucosal organs as diverse as the lung, kidney, and intestine, inevitably require the intimate interactions of neutrophils with columnar epithelia. The physiologic consequences of such interactions often determine endpoint organ function, and for this reason, much recent interest has developed in identifying mechanisms and novel targets for the treatment of mucosal inflammation. Elegant *in vitro* model systems incorporating purified human neutrophils and human epithelial cells grown in physiologic orientations have aided in discovery of new and insightful pathways to define basic inflammatory pathways. Here, we will review the recent literature regarding the interactions between columnar epithelial cells and neutrophils, with an emphasis on intestinal epithelial cells, structural aspects of neutrophil transepithelial migration, molecular determinants of neutrophil-epithelial cell interactions, as well as modulation of these pathways. These recent studies highlight the dynamic nature of these pathways and lend insight into the complexity of treating mucosal inflammation.

## BACKGROUND

Neutrophils (polymorphonuclear leukocyte, PMN) provide a primary defense against bacterial pathogens in our environment and are involved in inflammatory responses to these and other threats. Like most mucosal surfaces, PMN migration into the epithelium is thought to be a first line of innate defense against infectious agents, and defects in such PMN-epithelial interactions can contribute to fulminate infections, mucosal ulcerations, and delayed tissue healing. The protective aspects of PMN-epithelial interactions at such surfaces is exemplified by the clinical observation that patients with primary defects in PMN function support ongoing mucosal disease, including neutropenic patients, and patients afflicted with genetic PMN immunopathologies (e.g., Leukocyte Adhesion Deficiency, Chediak-Higashi syndrome, myeloperoxidase deficiency, etc.)[1,2,3]. As a corollary, extensive functional defects in PMN have also been observed in patients with rapidly progressive, prepubertal, and juvenile periodontitis[4].

Investigations into the understanding of structural and molecular aspects of PMN-epithelial interactions have been aided significantly by the dedicated development of elegant *in vitro* model systems. The complex nature of the intact mucosa prohibits addressing specific and focused questions regarding single cells / single cell populations. To circumvent this complexity of the intact mucosa, *in vitro* models chiefly rely on epithelial cells, often cell lines, grown in native orientation[5]. Originally, it was appreciated that, unlike vascular endothelia-PMN interactions, a limitation in studying PMN-epithelial interactions included the anatomical constraint that PMN migrate from the basolateral domain to the apical domain of polarized epithelia, thus requiring that epithelial cells be grown in an inverted orientation. Using modifications of commercially available permeable supports, monolayers of epithelial cells were grown in standard or inverted configurations[6], and PMN migration was studied in these orientations. Directed transepithelial neutrophil migration was stimulated by constructing transmonolayer gradients of chemotactic substances recognized by neutrophils. Useful for this purpose are n-formylated peptides such as f-Met-Leu-Phe (fMLP). Since prokaryotes, but not eukaryotes, n-formylate proteins, n-formyl-peptide gradients radiating from bacterial sites and detected by neutrophil surface n-formyl-peptide receptors are a classic means by which neutrophils track bacteria. Hence, transepithelial fMLP gradients effectively drive transepithelial migration of neutrophils in such *in vitro* models and may approximate the process *in vivo*. Studies comparing PMN trafficking across epithelia grown in different orientations revealed a number of important differences, including the finding that PMN migrate more efficiently in the physiologic orientation[6], that polarized expression of surface molecules can differentially modulate PMN migration, and that sided release of bioactive compounds often determines the extent of PMN movement[7]. Moreover, these model systems allowed access to both apical and basolateral domains of the epithelial surface, and this particular aspect allowed for assessment of epithelial physiological function (paracellular permeability, vectorial ion transport, etc.) and identified a number of important physiologic modifiers of these processes[8].

## STRUCTURAL FEATURES OF NEUTROPHIL TRANSLOCATION ACROSS EPITHELIA

It has become clear that transepithelial migration of PMN significantly alters epithelial function. Epithelial cells are classically viewed as serving two major functions: the vectorial transport of ions, nutrients, and water, and the ability to seal the lumen from the subepithelial space, often termed "barrier function". This latter capability is attributable to intercellular tight junctions, present as gaskets which circumferentially join epithelial cells at their apices. Tight junctions regulate the passive permeation of hydrophilic solutes through the "paracellular" space. Clinical evidence suggests that PMN transepithelial migration diminishes epithelial barrier function, and it has been speculated that such alterations in epithelial permeability could have deleterious effects on vectorial ion transport[9]. Moreover, PMN-mediated changes in permeability may provide a route of entry for molecules and/or microorganisms to the subepithelial space, thereby providing a mechanism for initiation and maintenance of inflammation.

The molecular events underlying rearrangements of tight junctions by transmigrating PMN remain insufficiently understood. Studies utilizing transmigration inhibitors and PMN from patients with chronic granulomatous disease (a disorder in which PMN lack the ability to generate reactive oxygen species) suggests that the mechanism by which PMN migrate across tight junctions is not through proteolysis or oxidant production[10,11], but likely involves mechanical impalement of the tight junction. Studies with epithelial monolayers cultured on permeable membrane supports as well as isolated human amniotic membrane have shown that transepithelial migration of PMN can be promoted by classic chemoattractants such as leukotriene $B_4$ ($LTB_4$), platelet activating factor (PAF) and n-formyl-peptides. Such studies have demonstrated that PMN

transmigrate via the paracellular pathway across intercellular tight junctions, and as such, alter epithelial permeability[8]. Some evidence indicates that at least one of the transmembrane proteins of the tight junction complex, occludin, may serve as a critical regulator of PMN trafficking through the paracellular space, and mutational analysis identified areas within the one of the extracellular domains important in regulating PMN transmigration[12]. Of interest in this regard, it was recently shown that in tissues derived from patients with inflammatory bowel disease (IBD), a disorder characterized by intestinal permeability changes and large numbers of PMN trafficking through the epithelium, occludin expression is selectively lost throughout intestinal epithelia[13]. Moreover, these studies revealed that other tight junction and adherens junction proteins were downregulated areas directly adjacent to PMN transmigration. Such findings indicate the likelihood that PMN-epithelial crosstalk pathways exist *in vivo* and are pathophysiologically relevant to diseases of mucosal inflammation.

This disruption of tight junctions by transmigrating PMN appears to be a reversible process, with recovery of barrier function within hours of insult[14,15]. In the case of high density PMN transmigration, wound closure is initiated by epithelial flattening and the extension of cytoskeletal-enriched lamellipodia at the leading edge. Apically localized circumferential rings of F-actin and myosin were found to encircle such wounds, suggesting final closure by a sphincter-like contraction[16]. In addition, some evidence suggests that this "resealing" event following impalement of epithelial tight junctions by PMN is enhanced by biochemical crosstalk between these cell types. For example, terminal steps of PMN transmigration may result in the release of soluble mediators (e.g., adenosine, see later) which through activation of epithelial receptors, may promote epithelial barrier properties, thus functioning as an innate resealing mechanism[17].

The recent discovery of junctional adhesion molecule (JAM) has provided the potential for new and important insight into paracellular trafficking of PMN through the epithelium. JAM is a ~35 kDa immunoglobulin gene superfamily member that consists of two V-type Ig domains which localizes to the subapical compartment of the endothelial and epithelial tight junction[18]. Recent studies addressing the structure/function of human JAM suggest a broader distribution, and interestingly, such expression includes cells of hematopoietic lineage[19,20]. *In vitro* studies have indicated that JAM antibodies block monocyte transendothelial migration[18], inhibit epithelial resealing following transient divalent cation depletion[20], and block reovirus invasion of epithelial monolayers[21]. *In vivo* studies in the mouse indicated that inhibitory antibodies directed against JAM blocks monocyte migration in the subcutaneous air pouch model[18] and inhibits leukocyte recruitment into the cerebrospinal fluid in experimental meningitis[22]. Further studies are necessary to define the exact role of JAM in regulation of leukocyte-epithelial interaction.

## MOLECULAR EVENTS GOVERNING NEUTROPHIL-EPITHELIAL INTERACTIONS

PMN migration across endothelia and epithelia is a result of an orchestrated series of timely events, ultimately resulting in PMN accumulation at sites of tissue injury (see Fig. 1). It is now appreciated that adhesion-based interactions, involving specific cell adhesion epitopes, are the primary means by which PMNs interact with other cell types, including endothelial cells[23,24,25] and epithelial cells[8,26]. For example, recent studies have shown that PMN $\beta_2$ integrins are required for PMNs to move across oral epithelia[27], renal epithelia[28], and intestinal epithelia[6,29,30]. These integrins, like others, are heterodimeric glycoproteins which exist in four forms on the PMN. Each displays a unique $\alpha$-subunit (CD11a, b, c, or d) and an identical $\beta$-subunit (CD18)[25,31]. These receptors are best demonstrated in the genetic disorder Leukocyte Adhesion Deficiency (LAD), in which patients lack normal expression of the CD 18 $\beta$-subunit, and as a result, show increased susceptibility to infection due to abnormal leukocyte

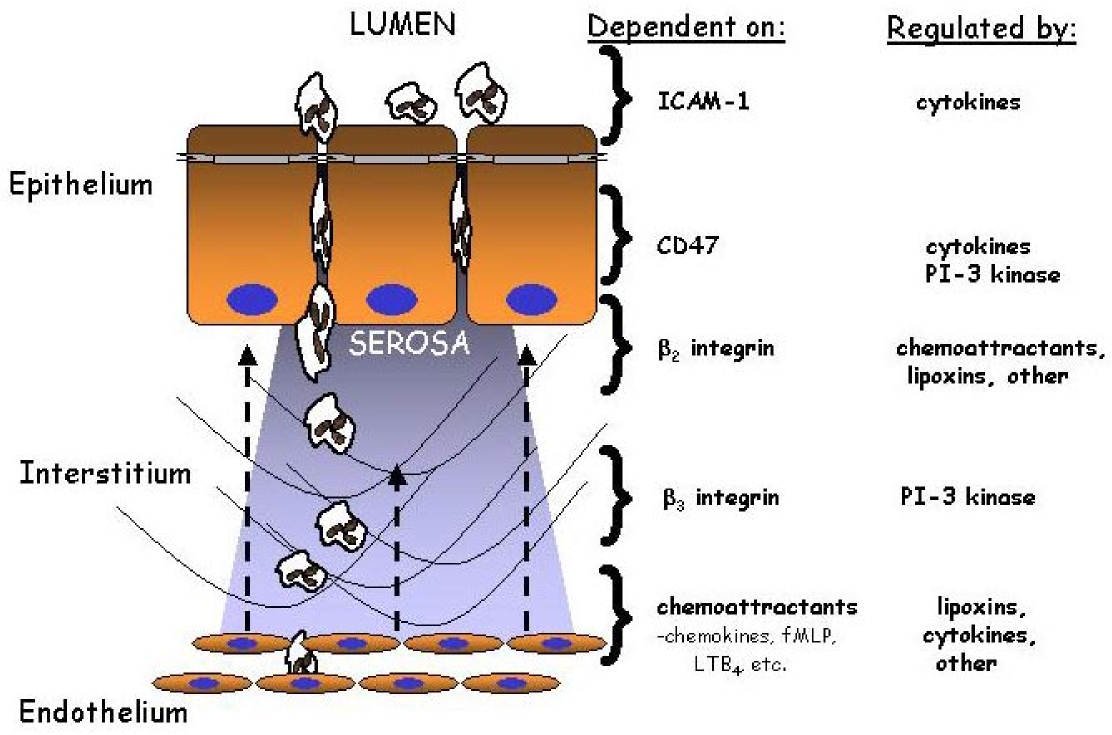

**FIGURE 1.** Multi-step model of neutrophil emigration into and across mucosal epithelial cells. This pathway can be conceptually divided into 5 interdependent events: (a) microvascular emigration, (b) transmigration across the lamina propria matrix, (c) initial interaction with the basal pole of epithelia, (d) transepithelial migration, and (e) functional interaction with the apical epithelial membrane. As indicated, these steps are dependent on surface or soluble molecules. A partial list of regulatory mechanisms are also listed.

function[25] A common clinical symptom of these patients is severe mucosal disease[1,32]. In unmodulated epithelia (i.e., cells which have no pre-exposure to inflammatory cytokines) CD11b/18 interactions of the PMN with the epithelial surface are required for transmigration, but CD11a or CD11c/18 interactions are not. Moreover, PMNs from LAD patients fail to migrate across intestinal epithelial monolayers[6,29,30], further indicating the dependence of this event on PMN expression of CD11/18 integrins. $\beta_2$ integrin-based interactions also occur between PMNs and endothelial cells, although integrin specificity varies in this system, suggesting that different rules govern transendothelial and transepithelial migration. The epithelial counter-receptor for PMN $\beta_2$ integrins remains elusive. Unlike endothelial cells, a number of studies utilizing diverse epithelial models have demonstrated that intercellular adhesion molecule-1 (ICAM-1) is not an epithelial ligand for PMN $\beta_2$ integrins[6,27,29,33,34], although under some conditions, ICAM-1 may function as an apical anchor for PMN (see Fig. 1)[34]. Functional mapping studies of this $\beta_2$ integrin-dependent pathway has suggested that the profile of inhibition is distinct from that of other known ligands of CD11b/CD18[35]. Such data suggest novel pathways for PMN trafficking across the epithelium, and discovery of such pathways provides a potential therapeutic target.

Studies directed at understanding specific PMN-epithelial interactive events unveiled a functionally inhibitory monoclonal antibody (mAb) which blocks PMN transmigration, but not PMN adhesion, to epithelia[17,33,36]. Subsequent experiments revealed that the antigen recognized by this mAb represents a membrane glycoprotein of ~60 kD and is expressed in a polarized fashion (basolateral) and was identified as CD47 (also termed integrin-associated protein), a previously cloned protein with homology to the immunoglobulin supergene family[37], with multiple ligands and a number of demonstrated leukocyte functions[38].

Similarly, others have demonstrated that CD47 is important in PMN transendothelial migration[39], suggesting some degree of universality for CD47 in leukocyte-mediated interactions. Of note, this CD47 pathway of transepithelial migration is likely regulated by a number of critical checkpoints. First, CD47 expression is regulated by at least one cytokine with a known regulatory role in PMN transepithelial migration (interferon-gamma[36]). Second, CD47 may function in a kinetic manner. For example, it was recently shown that while monoclonal antibodies directed against CD47 delay transepithelial migration, with time, this anti-CD47 influence is overcome[40]. Such surface CD47 function was linked to as yet undefined tyrosine phosphorylation events, suggesting a critical kinetic signaling pathway influencing the rate of PMN migration. Third, recent studies suggest that transmembrane CD47 signaling function may function, at least in part, in concert with signal-regulatory protein-alpha (SIRP$\alpha$), a cell surface protein containing three immunoglobulin superfamily domains and intracellular immunoreceptor tyrosine-based inhibitory motifs (ITIMs)[41]. CD47 is a ligand for Sirp$\alpha$, and studies have suggested that CD47 function is proportional to the expression of Sirp$\alpha$ and that Sirp$\alpha$ interactions with CD47 binding may mediate cell-cell interactions[38]. Thus, it appears that CD47 is critically important to regulated transepithelial migration.

Following successful migration across the vascular endothelia, PMN must maneuver the interstitium in route to epithelial surfaces. Matrix substrates may provide additional signaling pathways as directives for movement through this extracellular space. Recent studies have defined phosphoinositide 3-kinase (PI3K) as an important molecule in acute inflammatory processes. The p110$\gamma$ subunit of PI3K is the major PI3K activated during chemoattractant stimulation of PMN[42], and transgenic knockout mice lacking the p110$\gamma$ subunit manifest defects in a number of PMN responses, including protein kinase Akt activation, formation of 3'-phosphorylated lipids, superoxide anion generation, and chemotaxis[43,44,45]. Similarly, PMN migration across substrates laid down by epithelial cells (termed transmatrix migration), are substantially influenced by inhibitors of PI3K (e.g., wortmannin, which inhibits all forms of PI3K[46]). The mechanism(s) for PI3K inhibition of PMN migrations may lie, as least in part, in the regulation of intracellular $\beta_3$ integrin coupling[47] (see Fig. 2). Indeed, PMN-matrix interactions are potently inhibited by antibodies directed against $\beta_3$ integrins, and PI3K inhibitors block such $\beta_3$ integrin dependence. Biochemical analysis of intracellular $\beta_3$ integrin uncoupling by PI3K inhibitors revealed diminished $\beta_3$ integrin tyrosine phosphorylation and decreased association with $p72^{syk}$. Similarly, the $p72^{syk}$ inhibitor piceatannol promoted PMN transmatrix migration while HIV-tat peptide-facilitated loading of peptides corresponding to the $\beta_3$ integrin cytoplasmic tail identified the functional tyrosine residues for this activity. Similarly, it was recently shown that inhibition of PI3K increases the rate of PMN transmigration and rapidly upregulates PMN expression of CD47[40]. Taken together, these findings indicate that PI3K-regulated migratory pathways represent a endogenous "braking" mechanism for PMN during transit through the extracellular matrix en route to the basal pole of the epithelium.

## ROLE OF EPITHELIA IN ORCHESTRATION OF INFLAMMATION

PMN migration across endothelia and epithelia is a result of an orchestrated series of timely events, ultimately resulting in PMN accumulation at sites of tissue injury. *In vivo*, such an interplay often involves the contribution of numerous cell types in a coordinated fashion. For example, in the late 1960s, it was observed that mice colonized with pathogenic bacterial strains resulted in the massive accumulation of PMN at the epithelial surface[48]. Such morphologic observations have lead to a burgeoning area of active research into defining novel mechanism of bacterial-epithelial-PMN interactions[49]. While too numerous to review here, a few highlighted

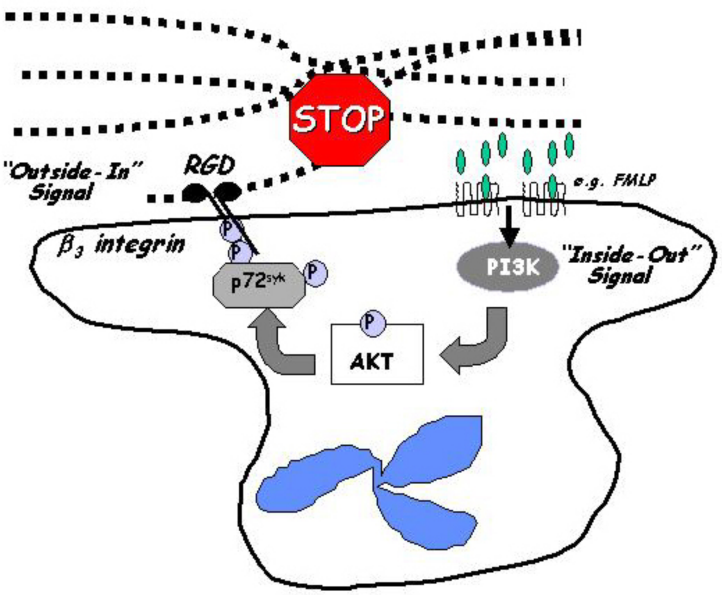

**FIGURE 2.** Biochemical model of endogenous "braking" system mediated by $\beta_3$ integrin interactions with the extracellular matrix**.** During PMN interactions with extracellular matrices, PMN activation through a "two-hit" mechanism ($\beta_3$ integrin binding to RGD-containing matrix liagands and chemotactic receptor activation, e.g., FMLP) suggests a combination of "outside-in" and "inside-out" pathways coupling $\beta_3$ integrin tyrosine phosphorylation and association with p72$^{syk}$. Central to this mechanism is PI-3 kinase-mediated phosphorylation of AKT.

studies provide important insight into basic mechanisms of inflammation. First, it is clear that pathogenic bacteria have evolved complex mechanisms to utilize host machinery to successfully invade and colonize the epithelium. The interaction of *Shigella* species with epithelial cells, for example, includes interactions of bacteria with the epithelial cell surface and release of Ipa proteins through a specialized type III secretion system. A complex signaling process involving activation of small GTPases of the Rho family and c-src results in dramatic rearrangements of the subcortical cytoskeleton. Actin-mediated motility promotes efficient colonization of the host cell cytoplasm and rapid cell-to-cell dissemination through protrusions that are engulfed by adjacent cells in a cadherin-dependent process[50]. Recent work suggests that blockade of *Shigella* interaction with the cytoskelton using the polyamine cadaverine severely abrogates epithelial signaling to PMN[51]. Second, successful bacterial invasion transforms infected cells into strongly proinflammatory cells, not the least of which includes the liberation of numerous factors for recruitment of large numbers of PMN to the epithelial surface. These factors include chemokines, cytokines, cell adhesion molecules, and lipid mediators[52], and some evidence indicates that PMN themselves may directly promote cytokine and chemokine induction in intestinal epithelia *in vivo*[53]. Third, some pathogenic bacterial strains have developed mechanisms to defend against host inflammatory elements. For example, studies addressing oral epithelial-PMN interactions have indicated that active bacterial infection (e.g., *Porphyromonas gingivalis*) may "paralyze" chemokine generation[54], and it has been proposed that this aspect may serve as a pathogenic mechanism for such oral pathogens[54]. Conversely, it was recently shown that some enteric organisms can function as to attenuate epithelial responses to prokaryotic organisms[55], providing insight into the unique tolerance of the mucosa to proinflammatory signals. Taken together, the dynamic interplay between bacteria and host epithelial cells may best exemplify the prominent role of epithelial cells in orchestration of inflammation.

It is now appreciated that PMN-mediated influences on epithelia, whether destructive or protective, do not necessarily require direct PMN-epithelial contact. Indeed, a number of soluble PMN-derived factors can directly influence epithelial function. Many PMN-derived compounds are released in response to PMN activation. At least two, and possibly three, distinct populations of granules exist within PMN. Release of soluble factors from one type of granule can occur independently of secretion from other types of granules[56]. The contents of primary and secondary granules are relatively well defined and include microbicidal enzymes, acid hydrolases, and various other proteins[57]. In addition, PMN also release compounds other than those present in granules; these include various metabolites (e.g., 5'AMP), lipids (e.g., platelet activating factor [PAF]), and membrane bound enzymes (e.g., phospholipases)[58,59]. Additionally, in response to activation by a number of stimuli, PMN may generate potent bactericidal reactive oxygen species[60]. Although various inflammatory stimuli may result in PMN degranulation, PMN activation (and transmigration) can occur without degranulation[61,62]. One of the better studied aspects includes the identification of PMN-derived 5'AMP as a mediator of epithelial function[58,63,64]. In this setting, 5'AMP is converted to adenosine (via ecto-5'-nucleotidase), and upon release into the extracellular milieu, adenosine is either taken up into the cell (via dipyridamole-sensitive carriers) or can interact with cell surface adenosine receptors[65]. At present, four subtypes of G protein-coupled adenosine receptors have been described, designated $A_1$, $A_{2a}$, $A_{2b}$, and $A_3$. These receptors are encoded by distinct genes and are pharmacologically differentiated by their relative affinities for adenosine analogs and methylxanthine antagonists[65]. Moreover, these receptors can be classified according to utilization of adenylate cyclase signal transduction pathways ($A_{2a}$ and $A_{2b}$) or pertussis toxin-sensitive pathways ($A_1$ and $A_3$)[65]. Epithelial cells of many origins express constitutive adenosine receptors[65]. Activation of these receptors results in a diverse set of actions, including activation of adenylate cyclase, induction of prostaglandin synthesis, and MAP kinase activation[66,67,68,69,70]. A recent study indicated that adenosine $A_{2b}$ receptor activation, such as occurs through PMN interactions with intestinal epithelial cells[58], results in the transcriptional induction of epithelial IL-6, which through paracrine mechansims, can modulate PMN functional responses[71].

## MODULATION OF PMN-EPITHELIAL INTERACTIONS

In the native mucosa, epithelial cells interface with the immune system via direct and indirect interactions with lymphoid cells[72]. As a result of these interactions, epithelial cell phenotypes vary according to the state of lymphoid activation. It is now well appreciated that epithelial cells are a source of, and express functional surface receptors for, a number of cytokines[73,74,75,76,77,78]. Ligation of surface cytokine receptors on epithelia have been shown to regulate barrier function, vectorial ion transport, expression of major histocompatibility antigens, as well as the interaction of epithelial cells with PMN[79]. This latter function is incompletely understood at present. As alluded to above, cytokines such as IFNγ, IL-1, and TNFα may regulate epithelial adhesion molecules[34,36,73,80], and epithelial chemokine synthesis is regulated by a number of cytokines[78,81]. Nonetheless, molecular details of cytokine-modulated PMN trafficking in the mucosa is an area in need of further study.

Epithelial surfaces such as the lung, intestine, and kidney support a rich and extensive underlying vasculature. Episodes of diminished blood flow, such as occurs with ischemia, vasculitis, or chronic inflammation, can result in significant tissue hypoxia[82], and neutrophils have a demonstrated role in tissue damage resulting from diminished blood flow and concomitant hypoxia[83]. The mechanisms underlying damage mediated by PMN under these conditions are complicated, and involve the generation of reactive oxygen intermediates, induction of cell-cell adhesion molecules and the liberation of a number of chemotactic factors[82,84]. Similarly, it is now appreciated that epithelial cells subjected to hypoxia are rich sources of proinflammatory cytokines/chemokines which can serve as directives for PMN trafficking into the epithelium[17,33,85,86]. Much recent attention has been paid to understanding the innate "dampening" mechanisms involved in inhibition of inflammation at mucosal sites. Of particular interest are a group of lipid mediators termed the lipoxins[87]. Lipoxins are bioactive eicosanoids derived from membrane arachidonic acid by the combined action of 5-lipoxygenase (LO) and 12-LO or 15-LO[88]. A number of recent *in vitro* and *in vivo* studies have revealed that lipoxins, and specifically lipoxin $A_4$ ($LXA_4$), functions as an innate "stop signal", functioning to control local inflammatory processes[89,90,91,92]. $LXA_4$ has been demonstrated to inhibit PMN transmigration across both endothelia and epithelia[90,91], block PMN diapedesis within the microcirculation of the hamster cheek pouch[93], and depress contraction of the guinea pig ileum[94]. Synthetic lipoxin analogs exhibit greater potency for these actions than the native compound, likely due to decreased metabolism to inactive compounds[91]. In particular, the synthetic $LXA_4$ analog 15 (R/S)-methyl-$LXA_4$, the structure of which resembles that of epimeric derivatives of lipoxin (15-epi-$LXA_4$), a native lipoxin generated *in vivo* in the presence of aspirin[89], may contribute in part to the anti-inflammatory actions of aspirin. Studies with human PMN *in vitro* indicate that 15 (R/S)-methyl-$LXA_4$ is a potent inhibitor of PMN transmigration across both endothelial and epithelial surfaces[95]. Similarly, the epi-lipoxin analogs have proven effective *in vivo*[96,97]. Thus, given their size (<400 daltons), stablility and composition, the lipoxins look especially promising as future therapeutic modalities for mucosal inflammation.

An additional aspect of eicosanoid metabolism was recently defined; that of inflammatory resolution via a switch from proinflammatory (e.g., leukotrienes and prostaglandins) to anti-inflammatory (e.g., lipoxins) lipid mediators. Such a switch occurred through temporal induction of 15-LO pathways via cyclic AMP responsive elements in the 15-LO gene and revealed that these functionally distinct lipid profiles drive PMN toward a program of inflammatory resolution[98]. Extensions of these findings could include other metabolic pathways than lipid mediators. For example, in the case of adenosine crosstalk pathways during PMN-epithelial interactions, a similar profile could result. Indeed, adenosine $A_{2b}$ receptor ligation results in elevations of intracellular cyclic AMP[63], which could feed-forward to induce/upregulate 15-LO expression and thus drive a resolution phase. Such mechanisms have not been considered until now, and these findings define a new paradigm for understanding the complexity of regulated inflammatory responses. Future work in these arena will define the precise role of individual cells types in the regulatory phases of ongoing inflammation.

## CONCLUSIONS

The dynamic interplay of PMN with epithelial surfaces defines a complex and elegant lesson in cell biology. Studies of model systems incorporating columnar epithelia cells and purified PMN have allowed for the identification of structural and functional determinants now well accepted in the scientific literature. The identification of epithelial determinants which bind to and associate with PMN will continue to define important information into the molecular regulation of cell-cell interactions, and such information may provide previously unappreciated insight into the pathogenesis of diseases associated with inflammation of surfaces lined by epithelia. Understanding of the mechanisms by which PMN alter epithelial ion transport may permit insights into new approaches to interfere with PMN activated events, such as fluid secretion by intestinal epithelia. As it is now well accepted that the rules governing epithelial-PMN interactions (and ultimately resulting to epithelial dysfunction) differ from those of PMN-endothelial interactions, applications of similar strategies may define more tissue specific therapeutic interactions.

## ACKNOWLEDGMENTS

This work was supported by grants RO-1 DK50189, F32 DE05762-01, and PO-1 DE13499 from the National Institutes of Health and by a grant from the Crohn's and Colitis Foundation of America.
